# Factors associated with wearing inadequate outdoor footwear in populations at risk of foot ulceration: A cross-sectional study

**DOI:** 10.1371/journal.pone.0211140

**Published:** 2019-02-21

**Authors:** Alex L. Barwick, Sheree E. Hurn, Jaap J. van Netten, Lloyd F. Reed, Peter A. Lazzarini

**Affiliations:** 1 School of Health and Human Sciences, Southern Cross University, Bilinga, Queensland, Australia; 2 School of Clinical Sciences, Queensland University of Technology, Brisbane, Queensland, Australia; 3 Institute of Health and Biomedical Innovation, Queensland University of Technology, Brisbane, Queensland, Australia; 4 Wound Management Innovation Cooperative Research Centre, Brisbane, Queensland, Australia; 5 Department of Rehabilitation, University of Amsterdam, Amsterdam Movement Sciences, Amsterdam, the Netherlands; 6 Footmotion Podiatry Brisbane, Queensland, Australia; 7 Allied Health Research Collaborative, Metro North Hospital & Health Service, Brisbane, Queensland, Australia; University of Illinois at Urbana-Champaign, UNITED STATES

## Abstract

**Background:**

Few studies have investigated if people at risk of foot ulceration actually wear the footwear recommended by best practice guidelines to prevent foot ulceration. This study aimed to investigate the prevalence of, and factors associated with, wearing inadequate outdoor footwear in those with diabetes or peripheral neuropathy in an inpatient population.

**Methods:**

This was a secondary analysis of a multi-site cross-sectional study investigating foot conditions in a large representative inpatient population admitted into hospital for any medical reason on one day. A range of explanatory variables were collected from all participants including sociodemographic, medical and foot condition factors. The outcome variable for this study was the self-reported outdoor footwear type worn most by participants outside the house in the year prior to hospitalisation. The self-reported footwear type was then categorised into adequate and inadequate according to footwear features recommended in guidelines for populations at risk of foot ulceration. Logistic regression identified factors independently associated with inadequate footwear in all inpatient participants, and diabetes and neuropathy subgroups.

**Results:**

Overall, 47% of a total of 726 inpatients wore inadequate outdoor footwear; 49% of the 171 in the diabetes subgroup and 43% of 159 in the neuropathy subgroup. Wearing inadequate outdoor footwear was independently associated (Odds Ratio (95% Confidence Interval)) with being female in the diabetes (2.7 (1.4–5.2)) and neuropathy subgroups (3.7 (1.8–7.9)) and being female (5.1 (3.7–7.1)), having critical peripheral arterial disease (2.5 (1.1–5.9)) and an amputation (0.3 (0.1–0.7)) in all inpatients (all, *p*<0.05).

**Conclusions:**

Almost half of all inpatients at risk of foot ulceration reported wearing outdoor footwear most of the time that did not meet recommendations for prevention. We found women were much more likely to wear inadequate footwear. More work needs to be done to increase the uptake of footwear recommendations in these populations to prevent foot ulceration.

## Introduction

Foot ulceration has major impacts on the physical, psychological and social functioning of individuals [1], and the healthcare expenditure of populations [2]. A variety of chronic illnesses including chronic kidney disease, cerebrovascular disease, and most commonly diabetes mellitus can result in the critical risk factors for the development of foot ulcers–peripheral neuropathy, peripheral arterial disease (PAD) and deformity [3–6]. Prevention of foot ulceration is paramount in maintaining independence, quality of life and reducing health care needs for people with these risk factors, especially peripheral neuropathy.

Up to half of all foot ulcers result from trauma that could have been prevented by wearing adequate footwear [7]. Inadequate footwear precipitates trauma via a number of different mechanisms, including: footwear lacking a protective enclosed upper allows acute external trauma; ill-fitting or non-fastening footwear facilitates chronic repetitive shear stresses and footwear unable to redistribute high plantar pressure areas facilitates chronic repetitive plantar pressures [7, 8]. These mechanisms are particularly problematic for people with diabetes or neuropathy, due to the inability to detect chronic or acute trauma to the foot and high plantar pressures associated with these diseases [9, 10]. Unfortunately, once a foot ulcer develops, intensive ongoing health care provision is required to facilitate healing and prevent the cascade of infection, hospitalisation, amputation and potentially death [2, 4].

For these reasons adequate preventative footwear is critical for people with diabetes and neuropathy and is a central recommendation in international guidelines for protecting feet from injury and preventing ulceration [11–14]. Along with recommendations for regular foot risk screening, daily self-inspection and treatment of any identified pre-ulcerative lesions, these guidelines strongly recommend wearing footwear with characteristics designed to adequately protect the foot from preventable trauma [11–14]. These recommended characteristics for adequate footwear include: i) appropriate size and shape to prevent chronic shear trauma, ii) fastenings to prevent chronic shear and plantar pressure trauma, iii) enclosed upper to prevent acute external trauma, iv) a shock absorbing sole to reduce chronic plantar pressure trauma, v) supportive heel counter to reduce chronic plantar pressure trauma, and vi) low heel elevation to prevent undue plantar forefoot pressure trauma [13, 14].

The limited research available in this field is highly inconsistent with a range of 14% to 91% of people with diabetes not wearing footwear with these recommended characteristics [8, 15–17]. Furthermore, the factors associated with wearing inadequate footwear in populations at risk of foot ulceration remain unknown. Such insights may provide a starting point for further research into cause and effect relationships and inform educational strategies to improve the wearing of adequate footwear in these at-risk populations in future. Thus, the aim of this study was to investigate the prevalence of, and factors associated with, wearing inadequate outdoor footwear in those with diabetes or peripheral neuropathy in an inpatient population.

## Materials and methods

### Study design

This study was a secondary analysis of data from the *Foot Disease in Inpatients Study*, a multi-site observational point-prevalence study with the aim of investigating foot-related conditions in a large inpatient sample considered highly representative of a typical population-based inpatient population [18, 19]. Ethics approval for this study was obtained from two Human Research Ethics Committee (HREC); The Prince Charles Hospital HREC (Ethics No. HREC/13/QPCH/5) and Queensland University of Technology HREC (Ethics No. 1300000367). Site specific authority was also obtained from each hospital and written informed consent was voluntarily obtained from all participants. The design and rationale of the study and measurement of variables are described in detail elsewhere [18, 19], and briefly below.

### Study population

All adult inpatients present in five public hospitals in Queensland (Australia) on one day were invited to participate; excluding those in a maternity or psychiatric ward or those with a cognitive deficit. Adult inpatients were defined as having been admitted to hospital for at least one night for any medical reason. Of 1,146 inpatients present on those days, 883 were eligible and 733 consented to participate. This sample of 733 participants (age 62±19 years, 55.8% male) has been reported to be highly representative of developed nations’ inpatient populations with respect to sociodemographic and medical history [18–20].

### Procedure

Trained data collectors surveyed each participant for their self-reported history and clinically examined their feet to diagnose foot-related conditions [18, 19, 21]. All data were captured on a data collection instrument (the Queensland Foot Disease Form) with a high degree of demonstrated validity and reliability [18, 19, 21].

### Explanatory variables

Self-reported explanatory variables included: sociodemographic factors (age, sex, indigenous status, country of birth, socioeconomic status, geographical remoteness), medical history (diabetes, previous foot ulceration, hypertension, dyslipidaemia, myocardial infarct, cerebrovascular accident, chronic kidney disease, cancer, arthritis, depression, smoking, mobility impairment, vision impairment), and foot treatment in the year prior to hospitalisation (by a podiatrist, general practitioner, specialist physician, surgeon, nurse, orthotist or other).

The clinically diagnosed explanatory variables included: lower extremity amputation history (performed during the current or previous admission), current foot ulceration, peripheral neuropathy, PAD and foot deformities. In brief, peripheral neuropathy was diagnosed as the failure to sense a 10-gram monofilament on at least two plantar forefoot sites on one foot. PAD was diagnosed when toe systolic pressure was <70mmHg. Severity of PAD was classified as mild (51-70mmHg), moderate (31-50mmHg) or critical (<30mmHg) [22, 23]. Foot deformity was diagnosed when three or more of the following characteristics were present on one foot: small muscle wastage, bony prominence, prominent metatarsal heads, hammer or claw toes, limited joint mobility or Charcot deformity [24].

### Outcome variable

The outcome variable of interest for this study was inadequate footwear type worn outside most of the time in the previous 12 months. Each participant was presented with a modified validated footwear picture chart [25] and asked to identify the footwear type that they wore the most often when they were not in their home. The chart displayed pictures of 16 different footwear types including: walking shoes, running shoes, oxford shoes, moccasins, boots, ugg boots, high heels, thongs/flip flops, slippers, backless slippers, court shoes, mules, sandals, bespoke footwear, socks only and was modified to add a barefoot (no footwear) option. The question asked, “from this chart displaying 16 different types of footwear, what is the one type of shoe you have worn most outside the house over the past 12 months?”. Although participants were in hospital, we did not consider it likely that any in-hospital footwear worn would have influenced responses as the average hospital stay in Australia is 4–5 days [20] and the question specifically asked for the footwear worn most over the past 12 months.

For the purposes of this study, footwear type was then collapsed into categories of ‘adequate’ and ‘inadequate’ based on international guideline recommendations for footwear for people with diabetes ulceration [11–14]. Adequate footwear was defined as those types with following features: i) wide toebox ii) fastening, such as laces or Velcro, iii) enclosed upper, iv) rubber or otherwise firm outsole v) firm heel counter, and vi) heel height of less than 2cm [13, 14]. Therefore, adequate footwear included the footwear types of walking shoes, running shoes, oxford shoes, boots and bespoke footwear. Inadequate footwear included all other footwear types: moccasins, ugg boots, high heels, thongs/flip flops, slippers, backless slippers, court shoes, mules, sandals, socks only and barefoot. Seven participants had missing footwear outcome data and were excluded from the study, leaving a total sample of 726 inpatient participants. Footwear adequacy was then investigated in three groups of interest: i) all inpatient participants, ii) participants with diabetes and iii) participants with neuropathy.

### Data analysis

All data were analysed using SPSS 22.0 for Windows (SPSS Inc., Chicago, IL, USA) or GraphPad Prism (GraphPad Software Inc., San Diego, CA, USA). Prevalence of adequate or inadequate footwear use (the outcome variable) was calculated along with 95% confidence intervals (95% CI) in all groups of interest (i.e. all inpatients, diabetes subgroup and neuropathy subgroup). Descriptive statistics were also calculated for each explanatory variable. For categorical variables, differences among the three groups were tested between groups using chi-squared tests with continuity correction or Fisher’s exact test if two cells had expected counts <5. For continuous variables, differences among the three groups were tested using Student’s t-tests (mean (standard deviation)) if normally distributed according to Kolmogorov-Smirnov tests. If continuous variables were not normally distributed, Mann-Whitney U tests (median (interquartile ranges)) were used to test differences.

Associations between explanatory variables and the outcome variable of footwear adequacy were examined using univariate logistic regression in all three groups of interest. All associations achieving a statistical significance of *p*<0.2 were included in backwards stepwise multivariate logistic regression analysis until only variables reaching statistical significance remained (*p*<0.05) (Unadjusted Model) [18, 26, 27]. The unadjusted model was then adjusted for age, sex, socioeconomic status and geographical remoteness by entering these variables into the model (Adjusted Model) [18, 26, 27]. Collinearity, goodness of fit, significance, parsimony and variance were assessed and found acceptable at each step [18, 26, 27]. Cases with missing data were excluded from all models as the proportion of missing data cases was minimal (<5% in all cases) [18, 26, 27].

## Results

Participant characteristics and univariate analyses for the three groups can be found in S1–S3 Tables. Prevalence (95% CI) of inadequate outdoor footwear worn was 46.8% (43.3–50.5%) in all inpatient participants (n = 726), 49.1% (41.7–56.6%) in the diabetes subgroup (n = 171) and 42.8% (35.3–50.5%) in the neuropathy subgroup (n = 159) (Table 1). There were no differences in the proportion of people who wore adequate outdoor footwear between diabetes and non-diabetes subgroups, or between neuropathy and non-neuropathy subgroups (*p*>0.2).

**Table 1:** Inadequate outdoor footwear worn in all groups.

	All n (%)	Diabetes n (%)	No diabetes n (%)	*p* Value	Neuropathy n (%)	No neuropathy n (%)	*p* Value
**Participants**	726	171	555		159	565	
**Adequate footwear**	386 (53.2%)	87 (50.9%)	299 (53.9%)	0.549	91 (57.2%)	295 (52.2%)	0.303
**Inadequate footwear**	340 (46.8%)	84 (49.1%)	256 (46.1%)	0.549	68 (42.8%)	270 (47.8%)	0.303

In the univariate analyses, wearing inadequate outdoor footwear in all inpatients was associated with being female, no history of amputation and PAD severity (all p<0.05). Wearing inadequate outdoor footwear in diabetes participants was associated with being female, no history of amputation and absence of neuropathy (all *p*<0.05). Wearing inadequate outdoor footwear in neuropathy participants was associated with being female and no history of amputation (all *p*<0.05).

In the multivariate analyses, after adjustment, we found that in all inpatients wearing inadequate outdoor footwear was independently associated (Adjusted Odds Ratio (95% CI)) with being female (5.1 (3.7–7.1)), amputation history (0.3 (0.1–0.7)) and critical PAD (2.5 (1.1–5.9)) (*p*<0.05) (Table 2). In the diabetes subgroup, after adjustment, wearing inadequate outdoor footwear was independently associated with being female (2.7 (1.4–5.2)) (*p*<0.005) (Table 3). In the neuropathy subgroup, after adjustment, wearing inadequate outdoor footwear was also independently associated with being female (3.7 (1.8–7.9)) (*p*<0.005) (Table 4).

**Table 2:** Independent factors associated with wearing inadequate outdoor footwear in all inpatients (Odds Ratios [95% CI]).

Risk Factor	Unadjusted	*p* Value	Adjusted^a^	*p* Value
**Female sex**	4.95 [3.59–6.83]	<0.001	5.10 [3.66–7.10]	<0.001
**Amputation history**	0.30 [0.12–0.75]	0.010	0.26 [0.10–0.68]	0.007
**PAD category**		0.019		0.028
**Nil**	1.00		1.00	
**Mild PAD**	0.52 [0.29–0.93]	0.028	0.60 [0.33–1.09]	0.095
**Moderate PAD**	1.40 [0.74–2.66]	0.304	1.48 [0.75–2.90]	0.256
**Critical PAD**	2.13 [0.96–4.74]	0.065	2.51 [1.07–5.91]	0.035
**Results:**	*Pseudo R*^2^^*b*^:*0*.*203* *Omnibus*^c^: *df* ^d^ *= 5*, *p <0*.*001*	*Missing*^e^: *4 (0*.*6%)* *H&L*^f^: *p = 0*.*813*	*Pseudo R*^2^^*b*^:*0*.*213* *Omnibus*^c^: *df* ^d^ *= 14*, *p <0*.*001*	*Missing*^e^: *25 (3*.*4%);* *H&L*^f^: *p = 0*.*721*

^a^Adjusted: for age, sex, socioeconomic status and geographical remoteness

^b^Pseudo R^2^: Nagelkerke R^2^

^c^Omnibus: Omnibus Tests of Model Coefficients

^d^df: degrees of freedom

^e^Missing: excluded missing cases

^f^H&L: Hosmer and Lemeshow Test.

**Table 3:** Independent factors associated with wearing inadequate outdoor footwear in diabetes participants (Odds Ratios [95% CI]).

Risk Factor	Unadjusted	*p* Value	Adjusted^a^	*p* Value
**Female sex**	3.02 [1.60–5.72]	0.001	2.66 [1.36–5.19]	0.004
***Results*:**	*Pseudo R*^*2*^^*b*^:*0*.*091* *Omnibus*^c^: *df* ^d^ *= 1*, *p <0*.*001*	^e^*Missing*: *0 (0%)* *H&L*^f^: *p = NA*^g^	*Pseudo R*^*2*^^*b*^:*0*.*127* *Omnibus*^c^: *df* ^d^ *= 10*, *p <0*.*001*	^e^*Missing*: *4 (2*.*3%);* *H&L*^f^: *p = 0*.*801*

^a^Adjusted: for age, sex, socioeconomic status and geographical remoteness

^b^Pseudo R^2^: Nagelkerke R^2^

^c^Omnibus: Omnibus Tests of Model Coefficients

^d^df: degrees of freedom

^e^Missing: excluded missing cases

^f^H&L: Hosmer and Lemeshow Test

^g^NA: Not applicable.

**Table 4:** Independent factors associated with wearing inadequate outdoor footwear in peripheral neuropathy participants (Odds Ratios [95% CI]).

Risk Factor	Unadjusted	*p* Value	Adjusted^a^	*p* Value
**Female sex**	3.03 [1.57–5.83]	0.001	3.73 [1.75–7.93]	0.001
***Results*:**	*Pseudo R*^*2*^^*b*^:*0*.*092* *Omnibus*^c^: *df*^*d*^ *= 1*, *p <0*.*001*	*Missing*^e^: *(0%)* *H&L*^f^: *p = NA*^g^	*Pseudo R*^*2*^^*b*^:*0*.*226* *Omnibus*^c^: *df*^*d*^ *= 10*, *p = 0*.*002*	*Missing*^e^: *5 (3*.*1%);* *H&L*^f^: *p = 0*.*890*

^a^Adjusted: for age, sex, socioeconomic status and geographical remoteness

^b^Pseudo R^2^: Nagelkerke R^2^

^c^Omnibus: Omnibus Tests of Model Coefficients

^d^df: degrees of freedom

^e^Missing: excluded missing cases

^f^H&L: Hosmer and Lemeshow Test

^g^NA: Not applicable.

## Discussion

We present novel data on the adequacy of footwear type worn most of the time outdoors prior to hospitalisation by populations at risk of foot ulceration and the factors associated with wearing inadequate outdoor footwear. We found almost half of the participants in the three groups studied (inpatients; participants with diabetes; participants with neuropathy) reported wearing a footwear type most of the time outside the house that did not meet guideline recommendations [11–14]. This suggests that those at risk of foot ulceration are not more likely to wear footwear recommended for prevention than those not at risk.

Previous studies that investigated footwear adequacy in diabetes populations have found varying rates of inadequate footwear use. Our finding that 49% of people with diabetes reported wearing inadequate outdoor footwear was comparable to the 47% found in a similar study in an Indian population [16]. However, the existing literature varies widely with findings of between 14% in a Canadian population [15] and 91% in a Filipino population with diabetes wearing inadequate footwear [17]. Climate and cultural factors may be responsible for this large variation. The types of footwear defined as adequate in these studies are all enclosed, which are more suitable and acceptable for the cooler climates seen in Canada whereas in warmer climates seen in Australia, India and the Philippines open shoes such as sandals and flip flops are more suitable, but defined as inadequate [12, 13].

In our study, we also found a significant gender difference towards adequate footwear with women more likely to wear inadequate footwear in all groups. This is similar to a study in a US diabetes population, where 27% of men and 55% of women wore inadequate footwear based on footwear characteristics in the previous 24 hours [8]. This indicates that women are consistently linked with wearing inadequate outdoor footwear even after adjusting for various sociodemographic, medical and foot condition factors. The reason for this difference may simply be that aesthetic considerations and sociocultural factors influence footwear choice along gender lines [28] as women’s footwear is more likely to display characteristics that do not reflect recommendations for adequate footwear to prevent foot ulceration, such as a higher heel height and smaller toe box [29]. The implication of this association is that women will be more vulnerable to footwear related ulceration. However, among those at risk, women experience rates of ulceration similar to that of men [30]. This is an interesting area for further research.

In our study, those with an amputation were almost four times less likely to wear inadequate outdoor footwear in inpatients. This may be due to a higher acceptance of the need to wear adequate footwear in this population to accommodate the amputated foot and to prevent further ulceration, especially after personally experiencing the negative outcomes of previous foot ulceration [31]. However, this relationship was not observed in the subgroups of diabetes and peripheral neuropathy. This might be explained by the relatively small number of people with amputations in these subgroups. In inpatients, we also found that those with critical PAD (toe pressure <30mmHg) were more likely to wear inadequate footwear. This may be because of limited self-care ability in people with critical PAD due to the significant concomitant cardiovascular disease as well as having different footwear priorities (e.g. warmth and comfort). However, again our finding could also be due to the small numbers of those with critical PAD. Future studies are required to investigate amputation history and critical PAD in larger at-risk populations to confirm our findings and discover potential reasons underlying the association.

Somewhat unexpectedly in all three groups there were no relationships (crude or independent association) identified between wearing inadequate outdoor footwear and other sociodemographic factors, medical history, past foot treatment factors or foot conditions. The lack of association with age, indigenous status, being born overseas, education levels, socioeconomic status or geographic remoteness suggests that use of inadequate outdoor footwear is prevalent across all sociodemographic categories. Even those who had foot treatment in the year prior to hospitalisation (and therefore more likely to have had footwear education on adequate footwear) were not less likely to wear inadequate footwear. This indicates that either health practitioners are not educating patients on wearing adequate footwear, or the education on adequate footwear is not successful at motivating footwear behaviour change.

The foot conditions of foot ulceration history and foot deformity were also not crudely or independently associated with wearing adequate outdoor footwear. This is in stark contrast to international guidelines explicitly recommending that adequate footwear is critical to accommodate foot deformities and prevent foot re-ulceration [11–14]. These findings emphasise the critical need for health practitioners to provide effective footwear education to people with diabetes and especially established neuropathy. More effective approaches to education that motivate and facilitate behaviour change in footwear practices are needed to aid in the prevention of adverse health outcomes in at-risk populations [32, 33].

There are some limitations to our study. First, it was a secondary analysis of data collected for a larger inpatient study and may not reflect the general population. However, the cohort has been shown to be highly reflective of a population-based inpatient population in developed nations [18, 19]. Second, due to the cross-sectional study design, inferences about the cause and effect relationship of the associations found cannot be made. We have interpreted some of the associations as being potentially causal within the context of previous research, however, these require further investigations. Third, although the multiple explanatory variables investigated have high validity and reliability [18–20], most were either self-reported or based on clinical diagnoses and not from gold standard invasive tests such as from pathology, nerve conduction studies or angiograms. Finally, the self-reported outcome variable of footwear “worn most outside the house in the previous year”, whilst based on a validated tool [34] has some obvious limitations. This self-reported selection may not be completely representative of the participants’ overall footwear use as it is reliant on the participant’s recall and only allowed for one footwear type to be selected. Other information about the adequacy of footwear, such as fit and shape, were not collected and it is known that people with diabetes often wear ill-fitting footwear [35]. Further, the categorisation of footwear into adequate and inadequate based on self-selected footwear type assumes that the individual shoe had the desired/undesired features typical of the footwear type. Overall, these limitations suggest our findings for inadequate outdoor footwear are most likely an underestimate.

## Conclusions

The findings from our study suggest that only half of those who need adequate footwear to prevent foot ulceration wear it, and women are much more likely to not wear adequate footwear. Additionally, those who have had foot care from a health professional in the previous year, were also not more likely to wear adequate footwear that meets international recommendations. More needs to be done to facilitate positive footwear changes to promote foot health and mobility in these populations to prevent foot ulceration. These findings should start to enable health professionals to acknowledge and address the challenges of wearing adequate outdoor footwear with their patients, especially in women.

## Supporting information

S1 Table Characteristics and univariate analysis for all inpatients wearing inadequate outdoor footwear. *p < 0.2; **p < 0.05; ^ 95% CI are for prevalence figure; GP: General Practitioner; IQR: Interquartile range; NA: Not applicable; PAD: Peripheral Artery Disease; SD: Standard deviation.
(DOCX)

S2 Table Characteristics and univariate analysis for diabetes participants wearing inadequate outdoor footwear. *p < 0.2; **p < 0.05; ^ 95% CI are for prevalence figure; GP: General Practitioner; IQR: Interquartile range; NA: Not applicable; PAD: Peripheral Artery Disease; SD: Standard deviation.
(DOCX)

S3 Table Characteristics and univariate analysis for peripheral neuropathy participants wearing inadequate outdoor footwear. *p < 0.2; **p < 0.05; ^ 95% CI are for prevalence figure; GP: General Practitioner; IQR: Interquartile range; NA: Not applicable; PAD: Peripheral Artery Disease; SD: Standard deviation.
(DOCX)
