# Supplementary material for: Factors associated with wearing inadequate outdoor footwear in populations at risk of foot ulceration: A cross-sectional study
Source: PLoS One. 2019 Feb 21;14(2):e0211140. doi: 10.1371/journal.pone.0211140 (PMC6383933; doi:10.1371/journal.pone.0211140)
Supplement: S1 Table — *p < 0.2; **p < 0.05; ^ 95% CI are for prevalence figure; GP: General Practitioner; IQR: Interquartile range; NA: Not applicable; PAD: Peripheral Artery Disease; SD: Standard deviation. (DOCX) [file pone.0211140.s001.docx]

**S1 Table:** Characteristics and univariate analysis for all inpatients wearing inadequate outdoor footwear

| Variables | All | Inadequate Footwear | | |
| --- | --- | --- | --- | --- |
|  |  | n (%) | Odds ratio [95% CI] | *p* Value |
| Participants^ | 726 | 340 (46.8%) |  |  |
| Socio-demographics |  |  |  |  |
| Age: Mean (SD) years | 62.0(18.7) | 61.2(19.4) | 1.00 [0.99-1.00] | 0.325 |
| Age: Median (IQR) years | 65(50-76) | 65(47-77) |  | 0.578 |
| Male sex | 403 (55.7%) | 121 (35.7%) | 0.20 [0.15-0.28] | <0.001** |
| Indigenous | 34 (4.7%) | 13 (3.8%) | 0.69 [0.34-1.41] | 0.310 |
| Born overseas | 161 (22.2%) | 70 (20.6%) | 0.84 [0.59-1.19] | 0.316 |
| <Year 10 Education Level | 390 (53.9%) | 187 (55.3%) | 1.12 [0.83-1.50] | 0.462 |
| Socioeconomic Status |  |  |  | 0.796 |
| Most disadvantaged | 101 (14.3%) | 51 (15.7%) | 1.00 |  |
| Second most disadvantaged | 157 (22.3%) | 74 (22.8%) | 0.87 [0.53-1.44] | 0.598 |
| Middle | 97 (13.8%) | 45 (13.8%) | 0.85 [0.49-1.48] | 0.564 |
| Second least disadvantaged | 238 (33.8%) | 108 (33.2%) | 0.81 [0.51-1.30] | 0.388 |
| Least disadvantaged | 112 (15.9%) | 47 (14.5%) | 0.71 [0.41-1.22] | 0.213 |
| Geographic Remoteness |  |  |  | 0.942 |
| Major city | 430 (61.0%) | 203 (62.5%) | 1.00 |  |
| Inner regional area | 152 (21.6%) | 69 (21.2%) | 0.93 [0.64-1.35] | 0.700 |
| Outer regional area | 66 (9.4%) | 29 (8.9%) | 0.87 [0.52-1.48] | 0.620 |
| Remote area | 30 (4.3%) | 13 (4.0%) | 0.86 [0.41-1.80] | 0.681 |
| Very remote area | 27 (3.8%) | 11 (3.4%) | 0.77 [0.35-1.60] | 0.515 |
| Medical condition history |  |  |  |  |
| Diabetes | 171 (23.6%) | 84 (24.7%) | 1.13 [0.80-1.59] | 0.492 |
| Hypertension | 354 (48.8%) | 171 (50.3%) | 1.12 [0.84-1.50] | 0.438 |
| Dyslipidaemia | 233 (32.1%) | 110 (32.4%) | 1.02 [0.75-1.40] | 0.888 |
| Myocardial Infarct | 145 (20.0%) | 68 (20.0%) | 1.00 [0.70-1.45] | 0.986 |
| Cerebrovascular Accident | 85 (11.7%) | 34 (10.0%) | 0.73 [0.46-1.16] | 0.180* |
| Chronic Kidney Disease | 88 (12.1%) | 43 (12.6%) | 1.10 [0.70-1.71] | 0.684 |
| Cancer | 171 (23.6%) | 78 (22.9%) | 0.98 [0.67-1.32] | 0.715 |
| Arthritis | 270 (37.2%) | 125 (36.8%) | 0.97 [0.72-1.31] | 0.824 |
| Depression | 189 (26.0%) | 100 (29.4%) | 1.39 [1.00-1.94] | 0.052* |
| Smoker | 104 (14.3%) | 43 (12.6%) | 0.77 [0.51-1.18] | 0.227 |
| Ex-Smoker | 300 (41.3%) | 134 (39.4%) | 0.86 [0.64-1.16] | 0.327 |
| Mobility impairment | 238 (32.9%) | 117 (34.5%) | 1.15 [0.84-1.56] | 0.391 |
| Vision impairment | 110 (15.2%) | 54 (15.9%) | 1.11 [0.74-1.66] | 0.627 |
| Past foot treatment |  |  |  |  |
| Yes | 252 (34.7%) | 122 (35.9%) | 1.10 [0.81-1.50] | 0.534 |
| Podiatry | 178 (24.5%) | 90 (26.5%) | 1.22 [0.87-1.71] | 0.251 |
| GP | 91 (12.5%) | 41 (12.1%) | 0.92 [0.59-1.43] | 0.716 |
| Surgeon | 35 (4.8%) | 14 (4.1%) | 0.75 [0.37-1.49] | 0.408 |
| Physician | 21 (2.9%) | 8 (2.4%) | 0.69 [0.28-1.69] | 0.418 |
| Nurse | 19 (2.6%) | 10 (2.9%) | 1.27 [0.51-3.16] | 0.608 |
| Orthotist | 4 (0.6%) | 2 (0.6%) | 1.14 [0.16-8.11] | 0.899 |
| Other | 9 (1.2%) | 5 (1.5%) | 1.43 [0.38-5.35] | 0.600 |
| Foot-related conditions |  |  |  |  |
| Amputation history | 34 (4.7%) | 9 (2.6%) | 0.39 [0.18-0.85] | 0.018** |
| Foot ulcer history | 87 (12.0%) | 42 (12.4%) | 1.07 [0.68-1.67] | 0.783 |
| Peripheral neuropathy | 159 (22.0%) | 68 (20.1%) | 0.82 [0.57-1.16] | 0.263 |
| Foot deformity | 157 (22.4%) | 72 (21.8%) | 0.94 [0.66-1.35] | 0.743 |
| PAD Severity |  |  |  | 0.035** |
| Nil PAD | 572 (79.0%) | 275 (81.4%) | 1.00 |  |
| Mild PAD | 69 (9.5%) | 21 (6.2%) | 0.47 [0.28-0.81] | 0.006** |
| Moderate PAD | 50 (6.9%) | 24 (7.1%) | 1.00 [0.56-1.78] | 0.992 |
| Critical PAD | 33 (4.6%) | 18 (5.3%) | 1.30 [0.64-2.62] | 0.471 |

**p* < 0.2; ***p* < 0.05; ^ 95% CI are for prevalence figure; GP: General Practitioner; IQR: Interquartile range; NA: Not applicable; PAD: Peripheral Artery Disease; SD: Standard deviation
