# Supplementary material for: Factors associated with wearing inadequate outdoor footwear in populations at risk of foot ulceration: A cross-sectional study
Source: PLoS One. 2019 Feb 21;14(2):e0211140. doi: 10.1371/journal.pone.0211140 (PMC6383933; doi:10.1371/journal.pone.0211140)
Supplement: S2 Table — *p < 0.2; **p < 0.05; ^ 95% CI are for prevalence figure; GP: General Practitioner; IQR: Interquartile range; NA: Not applicable; PAD: Peripheral Artery Disease; SD: Standard deviation. (DOCX) [file pone.0211140.s002.docx]

**S2 Table:** Characteristics and univariate analysis for diabetes participants wearing inadequate outdoor footwear

| Variables | Diabetes | Inadequate Footwear | | |
| --- | --- | --- | --- | --- |
|  |  | n (%) | Odds ratio [95% CI] | *p* Value |
| Participants^ | 171 | 84 (49.1%) |  |  |
| Socio-demographics |  |  |  |  |
| Age: Mean (SD) years | 65.1(16.8) | 62.5(19.3) | 0.98 [0.96-1.00] | 0.050* |
| Age: Median (IQR) years | 68(58-77) | 66.5(53-78.5) |  | 0.300 |
| Male sex | 102 (59.6%) | 39 (46.4%) | 0.33 [0.18-0.62] | 0.001** |
| Indigenous | 14 (8.2%) | 8 (9.6%) | 1.44 [0.48-4.34] | 0.517 |
| Born overseas | 33 (19.3%) | 15 (17.9%) | 0.83 [0.39-1.79] | 0.639 |
| <Year 10 Education Level | 99 (57.9%) | 47 (56.0%) | 0.86 [0.47-1.57] | 0.613 |
| Socioeconomic Status |  |  |  | 0.927 |
| Most disadvantaged | 24 (14.4%) | 13 (16.3%) | 1.00 |  |
| Second most disadvantaged | 42 (25.1%) | 19 (23.8%) | 0.70 [0.26-1.91] | 0.486 |
| Middle | 19 (11.4%) | 10 (12.5%) | 0.94 [0.28-3.14] | 0.920 |
| Second least disadvantaged | 61 (36.5%) | 29 (36.3%) | 0.77 [0.30-1.98] | 0.583 |
| Least disadvantaged | 21 (12.6%) | 9 (11.3%) | 0.64 [0.20-2.07] | 0.450 |
| Geographic Remoteness |  |  |  | 0.558 |
| Major city | 103 (61.7%) | 50 (62.5%) | 1.00 |  |
| Inner regional area | 32 (19.2%) | 16 (20.0%) | 1.06 [0.48-2.34] | 0.886 |
| Outer regional area | 19 (11.4%) | 6 (7.5%) | 0.49 [0.17-1.39] | 0.179 |
| Remote area | 3 (1.8%) | 2 (2.5%) | 2.12 [0.19-24.11] | 0.545 |
| Very remote area | 10 (6.0%) | 6 (7.5%) | 1.59 [0.42-5.97] | 0.492 |
| Medical condition history |  |  |  |  |
| Diabetes | -- | -- | -- | -- |
| Hypertension | 114 (66.7%) | 57 (67.9%) | 1.11 [0.59-2.10] | 0.746 |
| Dyslipidaemia | 87 (50.9%) | 41 (48.8%) | 0.85 [0.47-1.55] | 0.595 |
| Myocardial Infarct | 49 (28.7%) | 24 (28.6%) | 0.99 [0.51-1.93] | 0.981 |
| Cerebrovascular Accident | 20 (11.7%) | 9 (10.7%) | 0.83 [0.33-2.12] | 0.695 |
| Chronic Kidney Disease | 48 (28.1%) | 23 (27.4%) | 0.94 [0.48-1.82] | 0.844 |
| Cancer | 36 (21.1%) | 17 (20.2%) | 0.91 [0.44-1.90] | 0.797 |
| Arthritis | 74 (43.3%) | 33 (39.3%) | 0.73 [0.40-1.33] | 0.301 |
| Depression | 44 (25.7%) | 24 (28.6%) | 1.34 [0.67-2.67] | 0.404 |
| Smoker | 15 (8.8%) | 8 (9.5%) | 1.2 [0.42-3.48] | 0.733 |
| Ex-Smoker | 80 (46.8%) | 37 (44.0%) | 0.81 [0.44-1.47] | 0.481 |
| Mobility impairment | 69 (40.4%) | 37(44.0%) | 1.35 [0.73-2.50] | 0.333 |
| Vision impairment | 34 (19.9%) | 19 (22.6%) | 1.40 [0.66-2.99] | 0.380 |
| Past foot treatment |  |  |  |  |
| Yes | 100 (58.5%) | 52 (61.9%) | 1.32 [0.72-2.43] | 0.372 |
| Podiatry | 84 (49.1%) | 44 (52.4%) | 1.29 [0.71-2.38] | 0.403 |
| GP | 39 (22.8%) | 19 (22.6%) | 0.98 [0.48-2.00] | 0.954 |
| Surgeon | 16 (9.4%) | 6 (7.1%) | 0.59 [0.21-1.71] | 0.333 |
| Physician | 5 (2.9%) | 2 (2.4%) | 0.68 [0.11-4.19] | 0.680 |
| Nurse | 11 (6.4%) | 7 (8.3%) | 1.87 [0.53-6.70] | 0.326 |
| Orthotist | 1 (0.6%) | 1 (1.2%) | 0 | NA |
| Other | 0 | 0 | 0 | NA |
| Foot-related conditions |  |  |  |  |
| Amputation history | 20 (11.7%) | 5 (6.0%) | 0.30 [0.11-0.88] | 0.028** |
| Foot ulcer history | 43 (25.1%) | 22 (26.2%) | 1.12 [0.56-2.23] | 0.757 |
| Peripheral neuropathy | 74 (43.3%) | 29 (34.5%) | 0.49 [0.27-0.91] | 0.024** |
| Foot deformity | 51 (30.5%) | 25 (30.5%) | 1.00 [0.52-.192] | 0.989 |
| PAD Severity |  |  |  | 0.285 |
| Nil PAD | 111 (64.9%) | 56 (66.7%) | 1.00 |  |
| Mild PAD | 25 (14.6%) | 8 (9.5%) | 0.46 [0.18-1.16] | 0.100 |
| Moderate PAD | 23 (13.5%) | 13 (15.5%) | 1.28 [0.52-3.15] | 0.596 |
| Critical PAD | 12 (7.0%) | 7 (8.3%) | 1.38 [0.41-4.60] | 0.605 |

**p* < 0.2; ***p* < 0.05; ^ 95% CI are for prevalence figure; GP: General Practitioner; IQR: Interquartile range; NA: Not applicable; PAD: Peripheral Artery Disease; SD: Standard deviation
