# Supplementary material for: Factors associated with wearing inadequate outdoor footwear in populations at risk of foot ulceration: A cross-sectional study
Source: PLoS One. 2019 Feb 21;14(2):e0211140. doi: 10.1371/journal.pone.0211140 (PMC6383933; doi:10.1371/journal.pone.0211140)
Supplement: S3 Table — *p < 0.2; **p < 0.05; ^ 95% CI are for prevalence figure; GP: General Practitioner; IQR: Interquartile range; NA: Not applicable; PAD: Peripheral Artery Disease; SD: Standard deviation. (DOCX) [file pone.0211140.s003.docx]

**S3 Table:** Characteristics and univariate analysis for peripheral neuropathy participants wearing inadequate outdoor footwear

| Variables |  | Peripheral Neuropathy | | |
| --- | --- | --- | --- | --- |
|  |  | n (%) | Odds ratio [95% CI] | *p* Value |
| Participants^ | 159 | 68 (42.8%) |  |  |
| Socio-demographics |  |  |  |  |
| Age: Mean (SD) years | 70.0 (14.1) | 67.6 (15.6) | 0.98 [0.96-1.00] | 0.059* |
| Age: Median (IQR) years | 73(62-80) | 70(59.5-79) |  | 0.175 |
| Male sex | 92 (57.9%) | 29 (42.6%) | 0.33 [0.17-6.36] | 0.001** |
| Indigenous | 9 (5.7%) | 3 (4.4%) | 0.65 [0.16-2.72] | 0.558 |
| Born overseas | 29 (18.2%) | 11 (16.2%) | 0.78 [0.34-1.79] | 0.561 |
| <Year 10 Education Level | 91 (57.6%) | 37 (55.2%) | 0.85 [0.45-1.60] | 0.605 |
| Socioeconomic Status |  |  |  | 0.198* |
| Most disadvantaged | 24 (15.6%) | 14 (21.9%) | 1.00 |  |
| Second most disadvantaged | 34 (22.1%) | 17 (26.6%) | 0.71 [0.25-2.05] | 0.531 |
| Middle | 20 (13.0%) | 7 (10.9%) | 0.39 [0.11-1.31] | 0.127 |
| Second least disadvantaged | 56 (36.4%) | 20 (31.3%) | 0.40 [0.15-1.06] | 0.064 |
| Least disadvantaged | 20 (31.3%) | 6 (9.4%) | 0.31 [0.09-1.07] | 0.064 |
| Geographic Remoteness |  |  |  | 0.102* |
| Major city | 97 (63.0%) | 37 (57.8%) | 1.00 |  |
| Inner regional area | 32 (20.8%) | 19 (29.7%) | 2.37 [1.05-5.36] | 0.038 |
| Outer regional area | 10 (6.5%) | 1 (1.6%) | 0.18 [0.02-1.48] | 0.111 |
| Remote area | 9 (5.8%) | 4 (6.3%) | 1.30 [0.33-5.14] | 0.711 |
| Very remote area | 6 (3.9%) | 3 (4.7%) | 1.62 [0.31-8.46] | 0.566 |
| Medical condition history |  |  |  |  |
| Diabetes | 74 (46.5%) | 29 (42.6%) | 0.76 [0.40-1.43] | 0.395 |
| Hypertension | 92 (57.9%) | 39 (57.4%) | 0.96 [0.51-1.82] | 0.911 |
| Dyslipidaemia | 64 (40.3%) | 28 (41.2%) | 1.07 [0.56-2.03] | 0.837 |
| Myocardial Infarct | 37 (23.3%) | 16 (23.5%) | 1.03 [0.49-2.16] | 0.947 |
| Cerebrovascular Accident | 24 (15.1%) | 10 (14.7%) | 0.95 [0.39-2.29] | 0.906 |
| Chronic Kidney Disease | 32 (20.1%) | 14 (20.6%) | 1.05 [0.48-2.30] | 0.900 |
| Cancer | 44 (27.7%) | 18 (26.5%) | 0.90 [0.45-1.82] | 0.770 |
| Arthritis | 73 (45.9%) | 29 (42.6%) | 0.79 [0.42-1.50] | 0.475 |
| Depression | 41 (25.8%) | 20 (29.4%) | 1.39 [0.68-2.84] | 0.367 |
| Smoker | 18 (11.3%) | 10 (14.7%) | 1.79 [0.67-4.81] | 0.249 |
| Ex-Smoker | 65 (40.9%) | 24 (35.3%) | 0.67 [0.35-1.27] | 0.216 |
| Mobility impairment | 95 (59.7%) | 41 (60.3%) | 1.04 [0.55-1.98] | 0.903 |
| Vision impairment | 33 (20.8%) | 14 (20.6%) | 0.98 [0.45-2.13] | 0.964 |
| Past foot treatment |  |  |  |  |
| Yes | 85 (53.5%) | 37 (54.4%) | 1.07 [0.57-2.01] | 0.835 |
| Podiatry | 67 (42.1%) | 32 (47.1%) | 1.42 [0.75-2.69] | 0.278 |
| GP | 37 (23.3%) | 14 (20.6%) | 0.77 [0.36-1.63] | 0.490 |
| Surgeon | 18 (11.3%) | 7 (10.3%) | 0.84 [0.31-2.28] | 0.724 |
| Physician | 8 (5.0%) | 2 (2.9%) | 0.43 [0.08-2.20] | 0.310 |
| Nurse | 10 (6.3%) | 5 (7.4%) | 1.37 [0.38-4.92] | 0.634 |
| Orthotist | 3 (1.9%) | 1 (1.5%) | 0.66 [0.06-7.48] | 0.740 |
| Other | 1 (0.6%) | 1 (1.5%) | 0 | NA |
| Foot-related conditions |  |  |  |  |
| Amputation history | 26 (16.4%) | 6 (8.8%) | 0.34 [0.13-0.91] | 0.032** |
| Foot ulcer history | 50 (31.4%) | 19 (27.9%) | 0.75 [0.38-1.49] | 0.411 |
| Peripheral neuropathy | -- | -- | -- | -- |
| Foot deformity | 66 (42.6%) | 25 (37.9%) | 0.71 [0.37-1.37] | 0.309 |
| PAD Severity |  |  |  | 0.561 |
| Nil PAD | 96 (60.8%) | 45 (67.2%) | 1.00 |  |
| Mild PAD | 27 (17.1%) | 10 (14.9%) | 0.67 [0.28-1.60] | 0.365 |
| Moderate PAD | 21 (13.3%) | 7 (10.4%) | 0.57 [0.21-1.53] | 0.262 |
| Critical PAD | 14 (8.9%) | 5 (7.5%) | 0.63 [0.20-2.02] | 0.436 |

**p* < 0.2; ***p* < 0.05; ^ 95% CI are for prevalence figure; GP: General Practitioner; IQR: Interquartile range; NA: Not applicable; PAD: Peripheral Artery Disease; SD: Standard deviation
